# A Case of Pelvic Haematocele

**Published:** 1862-11

**Authors:** J. R. Lothrop


ART. II.—*A Case of Pelvic Haematocele.* *By* J. R. Lothrop, M. D.

September 22d, Mrs. W. was admitted to the hospital of the Sisters of Charity. She was about thirty-five years of age, had borne seven living children, and had miscarried twice. She stated that her illness had begun about two weeks previously, and had continued with intervals of seeming improvement to the time of her admission. At that time she complained of great pain in the lower part of the abdomen, bearing down of the rectum, difficulty in urinating, with a burning pain upon the passage of the urine; chills and sweating. Her pulse was 108 per minute, tongue heavily coated, face flushed and expressive of great suffering. Her pains she described as more severe than the pains of labor, and being as she supposed pregnant, having passed two menstrual periods, she thought she was about to miscarry. Upon examination of the abdomen, externally, a tumor was plainly perceptible above the pubes, centrally situated, while the uterus could be distinctly felt lying in front, and pushed forward upon the pubes. By examination per vaginum, a large tumor was found occupying the posterior pelvic space, and pressing forward so much that the finger was with difficulty passed in front between it and the pubes to reach the os-uteri. The tumor was plainly felt through the rectum. The bowels were constipated. For several days nothing was attempted beyond measures to allay pain and procure a free evacuation of the bowels. For this purpose morphia was given quite freely at first, and enemas of water employed. These seeming not to act with sufficient energy, laxative medicine was administered with satisfactory result. These measures had the effect to diminish the pain, almost entirely remove the pain and difficulty in urinating, and brought about a much more comfortable condition of the patient, as the pulse became less frequent, and she had much more sleep. But although her appearance improved and her suffering was much lessened, she still had at tim s, often at night, severe bearing down pains.

This state of things continued till September 30th, on which day, after a careful examination both by the vagina and rectum, fluctuation, which had hitherto not been perceived, was very evident by the introduction of a finger into the rectum and vagina at the same time. At this time also the tumor had descended, and was prominent in the vagina. Under these circumstances it was deemed advisable to puncture the tumor and let out the fluid contents, whether pus or blood. Accordingly a large sized trocar was passed into the vagina, the finger having been introduced as a guide, and passsd into the tumor, which gave but a slight resistance. Withdrawing the trocar a stream of liquid blood, having no unpleasant odor, followed. The quantity which flowed through the canula was about ten ounces, but it flowed quite freely after its removal, during the night, so that in all it amounted to at least a pint. Great relief followed the operation, and the night following the patient enjoyed for the first time almost for weeks, most refreshing sleep. The next day the patient passed to the care of my successor, Dr. Rochester, therefore I am unable to give the subsequent history.

The history of this case, previously to its admission to the hospital, may be briefly given. Mrs. W. had been traveling, and on her return home, while at Detroit, experienced a severe pain in the pelvic region when stooping suddenly to pick up something. Soon after she took passage on a steamboat for Buffalo. On the passage, the next day after this experience of pain, she had flowing, not however very great. This recurred at intervals ever after her arrival at Buffalo. About two weeks before she came to the hospital, and while she was flowing, she took cold by wetting her feet. She was soon after attacked by severe bearing down pains in the pelvis, which continued with intervals of more or less severity while she remained at home. During this time she had nausea and vomiting following soon after the attack of pain, chill, followed by heat, constipation and pain in passing urine. She had for the greater part of the time kept her bed.

In this case it is observable that the patient was thirty-five years of age, within the period of life when pelvic hæmatocele is most likely to occur. She had passed one or two menstrual periods. The access of pain, as far as can be gathered from her relation, though somewhat sudden, does not appear to have been attended with marked collapse, or if any, not by one which persisted. From this we infer that the extravasation was subperitoneal, rather than intra-peritoneal; that it was a pouring out of blood into the cellular tissue between the rectum and vagina, beneath the peritoneum.

The puncture was made through the vaginal walls in this case, because the tumor had pushed down lower into the vagina than into the rectum; in fact had the appearance of pointing in that direction, so that had spontaneous rupture taken place, appearances indicated that it would have opened into the vagina rather than the rectum. The selection of the vaginal surface is not to be taken as evidence of the preference of that to the rectal surface in all cases. There seem to be very good reasons for preferring the puncture through the rectum, viz: that spontaneous opening in the majority of cases is into it; that the discharge is more under control, and therefore less annoying, and that the most dependent portion of the tumor is usually there situated. There can be no danger of inflicting a wound upon the peritoneum, in a case of sub-peritoneal hæmatocele, because it is pushed upward out of reach, i. e. no danger which appertains to the one method more than the other. In a case of intra-peritoneal hæmatocele a puncture or incision either through the rectum or vagina, must equally wound the peritoneum, since it forms part of the sac of the tumor. So that any preference for one method over the other based upon a greater or less liability to wound the peritoneum, is practically without foundation, since it will wholly escape injury by either method, or be subjected to a wound, according as the extravasation has taken place above or beneath it.

Some writers speak of the probability of absorption in these cases. An extravasation to a limited amount, is probably absorbed, and this will account for the disappearance of tumors which have been discovered in the lower part of the abdomen. Obscure cases of sudden pain, resembling cramp in the pelvic region, accompanied by coldness and even collapse, in which no tumor has been observed and only some tenderness or sense of fullness, are capablc of this explanation, that a hæmorrhage to a limited amount has taken place from the uterus or its appendages which has subsequently undergone absorption. Is it proper then to delay interference, trusting to absorption for the removal of the tumor? In delaying we must be governed by the probabilities of absorption and by the dangers arising from making an opening. The former, in a large tumor, must be uncertain, even improbable, while the latter have not appeared to be specially great.

It seems certainly far better to make a free opening by which the contents of the tumor may be wholly evacuated, as then the continuance of the discharge is lessened, and of course will be less offensive in character. If a large sized trocar is sufficient to evacuate the tumor it is by far the most easily employed. The main object is to evacuate the contents of the tumor, and this object being secured it matters little whether it is by means of a trocar or an incision. There is no need of assuming that the trocar will fail to accomplish this, as instances prove the contrary, and that therefore an incision is in all cases preferable and necessary. The puncture by trocar is likely to secure the desired end, and if so, most men would probably think it most easy and simple, in the outset; should it not, an incision is then necessary. But it does not appear that peculiar advantages can be claimed for either proceeding, in the beginning.

